# A randomized trial of multivitamin supplementation in children with tuberculosis in Tanzania

**DOI:** 10.1186/1475-2891-10-120

**Published:** 2011-10-31

**Authors:** Saurabh Mehta, Ferdinand M Mugusi, Ronald J Bosch, Said Aboud, Anirban Chatterjee, Julia L Finkelstein, Maulidi Fataki, Rodrick Kisenge, Wafaie W Fawzi

**Affiliations:** 1 Division of Nutritional Sciences, Cornell University, Ithaca, NY 14853, USA; 2 Department of Internal Medicine, Muhimbili University of Health and Allied Sciences, Dar es Salaam, Tanzania; 3 Department of Biostatistics, Harvard School of Public Health, Boston, MA 02115, USA; 4 Departments of Microbiology and Immunology, Muhimbili University of Health and Allied Sciences, Dar es Salaam, Tanzania; 5 UNICEF, New York, NY, USA; 6 Department of Pediatrics, Muhimbili University of Health and Allied Sciences, Dar es Salaam, Tanzania; 7 Departments of Nutrition, Epidemiology, and Global Health and Population, Harvard School of Public Health, Boston, MA 02115, USA

## Abstract

**Background:**

Children with tuberculosis often have underlying nutritional deficiencies. Multivitamin supplementation has been proposed as a means to enhance the health of these children; however, the efficacy of such an intervention has not been examined adequately.

**Methods:**

255 children, aged six weeks to five years, with tuberculosis were randomized to receive either a daily multivitamin supplement or a placebo in the first eight weeks of anti-tuberculous therapy in Tanzania. This was only 64% of the proposed sample size as the trial had to be terminated prematurely due to funding constraints. They were followed up for the duration of supplementation through clinic and home visits to assess anthropometric indices and laboratory parameters, including hemoglobin and albumin.

**Results:**

There was no significant effect of multivitamin supplementation on the primary endpoint of the trial: weight gain after eight weeks. However, significant differences in weight gain were observed among children aged six weeks to six months in subgroup analyses (n = 22; 1.08 kg, compared to 0.46 kg in the placebo group; 95% CI = 0.12, 1.10; p = 0.01). Supplementation resulted in significant improvement in hemoglobin levels at the end of follow-up in children of all age groups; the median increase in children receiving multivitamins was 1.0 g/dL, compared to 0.4 g/dL in children receiving placebo (p < 0.01). HIV-infected children between six months and three years of age had a significantly higher gain in height if they received multivitamins (n = 48; 2 cm, compared to 1 cm in the placebo group; 95% CI = 0.20, 1.70; p = 0.01; p for interaction by age group = 0.01).

**Conclusions:**

Multivitamin supplementation for a short duration of eight weeks improved the hematological profile of children with tuberculosis, though it didn't have any effect on weight gain, the primary outcome of the trial. Larger studies with a longer period of supplementation are needed to confirm these findings and assess the effect of multivitamins on clinical outcomes including treatment success and growth failure.

**Clinicaltrials.gov Identifier:**

NCT00145184

## Introduction

*Mycobacterium tuberculosis *is one of the most successful pathogens known to man-both in terms of its longevity as well as in its ability to infect and cause disease in humans. Molecular genetics and genome sequencing techniques estimate that early forms of *M. tb *were present in East Africa at least 3 million years ago [1]; it remains the single most common curable infectious disease cause of mortality worldwide [2], despite the availability of effective anti-tuberculous chemotherapy. An estimated 250,000 children develop tuberculosis (TB) and 100,000 die of it every year worldwide [3].

Age and immune status of the child are two major determinants of progression to active TB after primary infection-the risk is highest in very young (< 2 years of age) and immunocompromised children [4,5]. Malnutrition and HIV infection increase this risk further [4,6,7]; for example, it is estimated that only one out of ten immunocompetent persons infected with TB develops active TB in his/her lifetime; whereas, one out of ten HIV-infected persons infected with TB will develop active TB every year [4].

Data from several studies indicate that TB is associated with weight loss and protein and calorie malnutrition [8-12] and such poor nutritional status in TB patients is a strong predictor of adverse events including treatment failure and mortality [13-17]. Studies among children without TB have shown a beneficial effect of multiple micronutrient supplementation on growth indices; for example, a meta-analysis showed that multiple micronutrient interventions improved linear growth (effect size: 0.09; 95% CI: 0.008, 0.17) [18]. In addition, micronutrient supplementation can also lead to boosting of the immune system, which may help improve the response to TB treatment. There is limited data on the prevalence of micronutrient deficiencies among children with tuberculosis in resource-limited settings; however, in a trial of multivitamin supplementation in Tanzania, 22% and 15% of children born to HIV-infected women, who did not receive multivitamin supplementation, were deficient in vitamins E (< 11.6 μmol/L) and B12 levels (< 150 pmol/L), respectively [19]. However, there are no studies of multivitamin supplementation among children with TB. In our previous work, we have also shown the benefits of maternal multivitamin (vitamins B-complex, C, and E) supplementation on child morbidity and mortality [20,21].

Therefore, we hypothesized that multivitamin supplementation would improve weight gain, a predictor for future growth and adverse clinical outcomes, in children with TB. To test this hypothesis, we conducted a randomized placebo-controlled trial among children with TB, both with and without HIV infection, in Dar es Salaam, Tanzania.

## Materials and methods

### Study Design and Population

**Figure 1:**
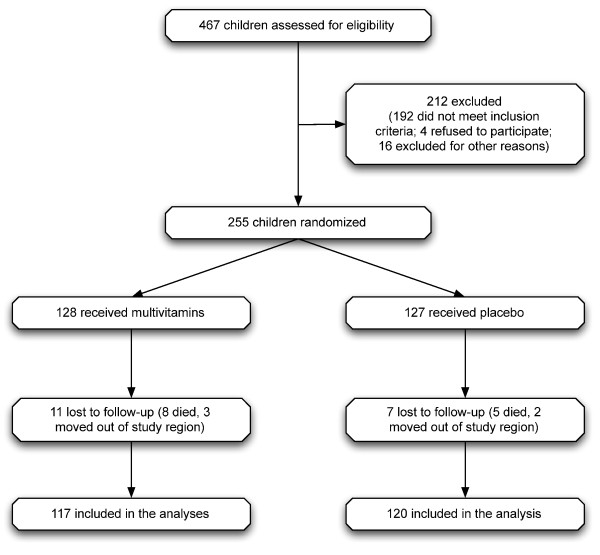
**Trial Profile**.

This study was a randomized double-blind placebo controlled trial among 255 children between the ages of six weeks and five years with probable tuberculosis. A total of 467 children aged six weeks to five years attending the pediatric clinic between May 2005 and September 2007 at the Muhimbili National Hospital in Dar es Salaam, Tanzania, were screened for signs and symptoms of TB (Figure 1). The inclusion criteria comprised of presenting with cough or wheezing for at least four weeks, fever of unknown origin, painless swelling in a group of cervical lymph nodes, loss of more than 10% of maximum weight, failure to gain weight for two months, or a history of household contact with a case of probable/confirmed TB in the past six months, and these children were diagnosed as having suspected TB. Children who had received anti-TB treatment for more than 4 weeks in the past year were not eligible.

A chest X-ray (Postero-Anterior view) was also obtained and a tuberculin skin test (TST) was conducted in all children. The TST used the standard WHO-purified protein derivative (PPD) and it was read after 48-72 hours. Children with a positive TST (induration greater than or equal to 10 mm in HIV-uninfected and 5 mm in HIV-infected) or with a chest X-ray indicative of TB (based on unequivocal lymphadenopathy or miliary TB) were categorized as probable TB (*n *> 275) and became eligible for enrolment in the study. The chest X-rays were read both by the study radiologist and the study pediatrician. Any discrepancies were resolved by a joint review of the radiological findings.

Trained research assistants obtained informed consent from the parents or guardians of the children in two stages-first, for eligibility testing and the second for participation in the trial.

Children with probable TB, written consent from parent/guardian, and no intent to leave the Dar es Salaam area over the next eight weeks were then randomly assigned to receive daily multivitamin supplements or placebo for the next two months (*n *= 255). An off-site statistician generated the randomization sequence; a list from 1 to 400 was prepared according to this randomization sequence in blocks of 20. At enrolment, each eligible child was assigned to the next numbered bottle of regimen at the site by the study staff. To minimize the risk of unblinding, the regimen bottles, with no visual difference between active regimen capsules and placebo capsules, were received from the manufacturer (Nutriset, France) without any identification; the study staff then labeled the bottles with the patient's initials and identification number. Both the clinicians and the patients were blinded to the study regimen, and the randomization list was kept confidential by the statistician, with the exception of the pharmaceutical company preparing the blinded treatment.

Each multivitamin capsule contained vitamins B_1 _0.5 mg, B_2 _0.6 mg, Niacin 4 mg, B_6 _0.6 mg, Folate 130 μg, B_12 _1 μg, C 60 mg, and E 8 mg. This composition was selected based on the demonstrated benefits of maternal multivitamin (vitamins B-complex, C, and E) supplementation on child morbidity and mortality [20,21], particularly in subgroups of children born to women who were immunologically or nutritionally compromised. Zinc and iron, on the other hand, were excluded because of concerns related to their supplementation, particularly among HIV-infected populations [22,23]. The roles of zinc and iron need to be examined separately rather than as part of a multi-nutrient regimen. Similarly, there is limited data on the utility of nutrients such as selenium and vitamin K in child health, and therefore, they were not included in the study regimen.

Children younger than six months in both the treatment and placebo groups received one capsule daily, whereas children between 7-36 months of age received 2 capsules daily, and children older than 36 months received three capsules daily. The doses provided were in multiples (two to five times) of the recommended dietary allowance as have been used in our earlier studies. Ampoules of sterile water to dissolve the contents of the capsule were also given to the study participants. Both placebo and the active regimen had a sweet taste when dissolved in sterile water. In addition, all children received standard anti-TB treatment, according to the guidelines of the National Tuberculosis and Leprosy Control Programme of Tanzania. The guidelines at the time of the trial recommended a six-month course of anti-tuberculous drugs (Isoniazid 50 mg, Rifampicin 200 mg, Ethambutol 10-15 mg/kg, and Pyrazinamide 20-30 mg/kg daily for 2 months, followed by Isoniazid 50 mg and Rifampicin 200 mg daily for 4 months) using Directly Observed Therapy (DOT).

The institutional review boards at Harvard School of Public Health, Boston, MA and Muhimbili University of Health and Allied Sciences, Dar es Salaam, Tanzania approved the study protocol.

### Baseline Assessment

At randomization, a trained research assistant obtained information on socio-economic and demographic characteristics. Weight, height/length, head circumference, mid-upper arm circumference (MUAC), and triceps skin-fold thickness, were measured by a study nurse according to standard methods. A physician also obtained a complete medical history and conducted a physical examination of the participating child at this visit.

### Follow-up procedures

All study subjects were followed up every two weeks; the study nurse visited the child at home during weeks 2 and 6 and the child was seen at the study clinic during weeks 4 and 8 of the study. During each visit, the study nurse enquired about the health of the child during the preceding two weeks and checked for compliance with anti-TB therapy and the study regimen through direct questioning of the parent/guardian and counting of the capsules left. During the clinic visits, the study nurse determined the anthropometric measurements, and a physician examined the child.

### Laboratory Methods

Venous blood was obtained from the participating children at entry into the study and at the last visit at 8 weeks after starting anti-TB therapy. Complete blood counts, including hemoglobin concentration, and albumin levels (using Roche Cobas Integra 400 plus analyzer) were determined for all participants. Total white blood cell count was measured using a Beckman Coulter AcT Diff II hematology analyzer and differential white blood cell count was also determined automatically. Absolute counts of T-cell subsets were measured using the FACSCount system (Becton-Dickinson, San Jose, CA).

HIV-1 (referred to as HIV henceforth) status was assessed through a double ELISA for children older than 18 months; any discrepancies were resolved using a Western blot test. For children younger than 18 months of age, HIV status was determined through Amplicor HIV-1 DNA PCR test version 1.5 (Roche Diagnostics, Branchburg, NJ).

### Statistical Analysis

The study was designed to enroll 400 children with TB and was powered (power = 80%) to detect a 27% difference in the primary endpoint of weight gain between the supplemented and the placebo groups. Due to funding constraints, we could not extend the enrollment period to increase sample size beyond 255. The attained sample size had adequate power to detect a 32.5% difference in weight gain between the supplemented and the placebo groups. Intention to treat analyses for all endpoints was used.

Descriptive statistics were expressed as the median with the first and third quartiles (interquartile range, IQR) and non-parametric Wilcoxon test was used for comparative statistics. Hodges-Lehmann 95% confidence limits were used to express the effect size of the difference between the two groups. We also examined the effect of the supplements within strata of age (younger than six months, seven-36 months, and older than 36 months) and HIV status. Tests of interaction were based on comparing the non-parametric estimate of the treatment effect [24] between strata, using their standard errors [25]. SAS version 9.2 (SAS Institute, Cary, NC) was used for all the analyses.

## Results

The baseline characteristics of the children enrolled in the trial are presented by treatment arm in Table 1. There were no significant differences between the two treatment arms. The median age was 18 months in both the placebo and the supplement groups. Forty seven percent and 44% of the children enrolled were females in the placebo and the supplemented groups, respectively. Twenty nine percent of the children in the placebo group and 39% in the supplemented group were HIV-infected. Only 3 children out of the 146 who had available gastric aspirate or sputum for culture had mycobacteria isolated in the sample.

**Table 1:** Baseline Characteristics of children with TB randomized in the trial (n = 255)

*Variable *	Placebo (n = 127)	Multivitamins (n = 128)
Age, in years, Median (IQR)^a^	1.50 (0.83, 2.42)	1.54 (0.79, 2.92)
Shillings^b ^spent on food per household per day, Median (IQR)^a^	2500.00 (2000.00, 3000.00)	2000.00 (2000.00, 3000.00)
Age categories, *n *(%)		
≤ 6 months	9 (7.09%)	13 (10.16%)
6 months to ≤ 3 years	93 (73.23%)	85 (66.40%)
> 3 years	25 (19.68%)	30 (23.44%)
Females, *n *(%)	60 (47.24%)	56 (43.75%)
HIV-infected, *n *(%)	37 (29.13%)	50 (39.06%)
≤ 6 months	4 (44.44%)	4 (30.77%)
6 months to ≤ 3 years	23 (24.73%)	29 (34.12%)
> 3 years	10 (40.00%)	17 (56.67%)
Marital Status of Mother, *n *(%)		
Single	15 (11.81%)	15 (11.72%)
Married	81 (63.78%)	70 (54.69%)
Divorced	2 (1.58%)	3 (2.34%)
Cohabiting	20 (15.75%)	32 (25.00%)
Separated	6 (4.72%)	7 (5.47%)
Widowed	3 (2.36%)	1 (0.78%)
Mother Literate, *n *(%)	114 (89.76%)	117 (91.41%)
Mother's Occupation, *n *(%)		
Housewife	75 (59.05%)	71 (55.47%)
Small business/Farm/Informal Income	41 (32.28%)	42 (32.81%)
Business woman	3 (2.36%)	6 (4.69%)
Public House/Restaurant	1 (0.79%)	1 (0.78%)
Professional (Nurse/Teacher)	2 (1.58%)	2 (1.56%)
Skilled Office Work	0 (0.00%)	2 (1.56%)
Unskilled Employment	3 (2.36%)	4 (3.13%)
Other	2 (1.58%)	0 (0.00%)
Meat or fish consumption, *n *(%)		
Never/Less than once per month	2 (1.61%)	4 (3.15%)
Sometimes/1-3 times per month	13 (10.48%)	11 (8.66%)
About once per week	33 (26.61%)	31 (24.41%)
2-4 times per week	69 (55.65%)	77 (60.63%)
Everyday/5-7 times per week	7 (5.65%)	4 (3.15%)
Admitted to the Hospital, *n *(%)	21 (16.54%)	21 (16.41%)

^a ^IQR: Inter-Quartile Range

^b ^1000 Tanzanian Shillings ≈ 1 US Dollar at the time of the trial

There were no significant differences in weight gain or changes in height, MUAC, head circumference, and triceps skin-fold thickness between the two groups during follow-up (Table 2). Children in both the placebo and the supplement groups gained a median of 0.84 kg in the two months since starting anti-TB therapy (*n *= 237; 95% CI = -0.15, 0.18; p = 0.82). However, there was significant effect modification by age group (p < 0.01); children younger than six months of age who received multivitamins gained a median of 1.08 kg, compared to 0.46 kg gained by children receiving placebo in that age group (95% CI = 0.12, 1.10; p = 0.01).

**Table 2:** Effect of multivitamin supplementation on anthropometric measurements

*Outcome *	*Time Point (n)^a ^*	Placebo	Multivitamins	Hodges-Lehmann	p-value^c ^
		Median (IQR)^b ^	Median (IQR)^b ^	95% CI	
				*(Multivitamin-Placebo)*	
Weight, kg					
	Baseline (255)	7.95 (6.20, 10.75)	8.48 (6.52, 10.81)	(-1.15, 0.36)	0.31
	Intermediate (245)	8.52 (6.84, 11.43)	9.38 (7.08, 11.81)	(-0.35, 1.28)	0.30
	Final (237)	9.01 (7.20, 12.02)	9.80 (7.74, 11.94)	(-0.38, 1.31)	0.28
	Change (237)	0.84 (0.45, 1.30)	0.84 (0.51, 1.30)	(-0.15, 0.18)	0.82
Height, cm					
	Baseline (255)	74.00 (67.50, 87.00)	77.50 (68.00, 86.55)	(-4.60, 1.00)	0.28
	Intermediate (245)	75.00 (68.00, 87.00)	77.45 (69.40, 88.00)	(-1.00, 4.80)	0.23
	Final (236)	76.00 (69.00, 88.20)	79.00 (70.00, 88.30)	(-1.00, 5.00)	0.24
	Change (236)	1.10 (0.50, 2.00)	1.50 (0.50, 2.50)	(-0.20, 0.50)	0.53
MUAC, cm					
	Baseline (255)	13.00 (11.20, 14.00)	13.00 (11.90, 14.25)	(-0.70, 0.25)	0.35
	Intermediate (245)	13.50 (12.00, 14.50)	14.00 (12.50, 15.00)	(-0.10, 0.80)	0.18
	Final (237)	13.80 (12.55, 15.00)	14.00 (12.70, 15.00)	(-0.30, 0.50)	0.56
	Change (237)	1.00 (0.20, 1.50)	0.80 (0.20, 1.50)	(-0.35, 0.20)	0.61
Head Circumference, cm					
	Baseline (254)	45.50 (43.00, 48.00)	46.00 (43.50, 48.00)	(-1.00, 0.50)	0.58
	Intermediate (245)	46.00 (43.80, 48.00)	46.45 (44.00, 48.00)	(-0.50, 1.00)	0.59
	Final (236)	46.00 (44.10, 48.50)	46.50 (44.40, 48.50)	(-0.50, 1.00)	0.58
	Change (235)	0.50 (0.00, 1.00)	0.50 (0.00, 1.00)	(-0.20, 0.25)	0.86
Triceps skin-fold thickness, cm					
	Baseline (255)	6.20 (5.20, 7.90)	6.15 (5.15, 8.00)	(-0.60, 0.40)	0.55
	Intermediate (245)	7.10 (5.60, 8.20)	7.20 (6.10, 8.95)	(-0.20, 0.90)	0.25
	Final (237)	7.50 (6.20, 8.60)	7.20 (6.00, 8.90)	(-0.70, 0.40)	0.61
	Change (237)	1.05 (-0.20, 2.10)	0.70 (-0.30, 1.90)	(-0.70, 0.20)	0.37

^a ^Intermediate is at 4 weeks follow-up; Final at 8 weeks follow-up; Change between the final and the baseline measurement

^b ^IQR: Interquartile Range

^c ^p-value based on Wilcoxon 2-group comparison

There was no difference in the gain in length/height during follow-up between the two groups (p = 0.53). However, HIV-infected children between the ages of 6 months and 3 years gained a median of 2 cm when receiving multivitamins, compared to 1 cm when receiving placebo (n = 48; 95% CI = 0.20, 1.70; p = 0.01; p for interaction by age group = 0.01).

The median increase in MUAC was 1.0 cm in the placebo group and 0.8 cm in the supplement group (p = 0.61). Head circumference increased by a median of 0.5 cm in both the placebo and the supplement groups (p = 0.86). The change in triceps skin-fold thickness over the period of follow-up was an 1.1 cm increase in the placebo group and 0.7 cm increase in the supplement group (p = 0.37).

There was no difference in the clearance of chest x-ray or mortality in the two treatment arms. 199 children had a chest X-ray available at the end of follow-up and 100 of them showed complete resolution. 13 children died during the course of the trial.

There was no significant difference in the hemoglobin levels in children in the placebo and supplement groups at baseline (Table 3); however, a significant difference was noted by the end of follow-up (*n *= 223) with the children in supplemented group having a greater increase in the hemoglobin levels by a median of 1.0 g/dL, compared to children in the placebo group (median of 0.4 g/dL; p < 0.01). In subgroup analyses by sex, this effect on hemoglobin was not statistically significant in female children (p = 0.11).

**Table 3:** Effect of multivitamin supplementation on laboratory parameters

*Outcome *	*Time Point (n)^a ^*	Placebo	Multivitamins	Hodges-Lehmann	p-value^c ^
		Median (IQR)^b ^	Median (IQR)^b ^	95% CI	
				*(Multivitamin-Placebo)*	
Hemoglobin, g/dL					
	Baseline (251)	8.70 (7.70, 9.80)	8.40 (7.40, 9.50)	(-0.20, 0.60)	0.28
	Final (225)	9.40 (8.45, 10.25)	9.60 (8.70, 10.60)	(0.00, 0.70)	0.05
	Change (223)	0.40 (-0.30, 1.40)	1.00 (0.30, 2.30)	(0.30, 1.00)	0.0001
Albumin, g/L					
	Baseline (253)	33.95 (29.90, 38.90)	34.00 (29.80, 39.10)	(-1.70, 1.70)	0.92
	Final (232)	40.20 (36.30, 42.90)	38.90 (33.80, 42.50)	(-2.70, 0.10)	0.08
	Change (231)	4.55 (0.10, 9.60)	3.40 (0.60, 7.00)	(-2.60, 0.60)	0.21
CD4, cells/μL					
	Baseline (242)	1592.50 (1099.00, 2254.00)	1351.50 (803.00, 2208.50)	(-410.00, 45.00)	0.11
	Final (212)	1507.50 (1038.50, 2011.50)	1410.50 (782.50, 1904.50)	(-373.00, 71.00)	0.19
	Change (207)	-63.00 (-506.00, 240.00)	-59.50 (-448.00, 261.00)	(-204.00, 140.00)	0.73
CD8, cells/μL					
	Baseline (242)	1233.00 (902.00, 2230.00)	1492.00 (988.00, 2624.00)	(-16.00, 450.00)	0.06
	Final (212)	1328.00 (921.50, 2208.50)	1506.50 (1039.00, 2742.00)	(-98.00, 389.00)	0.24
	Change (207)	-130.00 (-489.00, 394.00)	-77.50 (-735.00, 432.00)	(-322.00, 193.00)	0.68
CD3, cells/μL					
	Baseline (242)	3317.50 (2423.00, 4616.00)	3593.50 (2430.00, 5211.50)	(-311.00, 590.00)	0.53
	Final (212)	3176.00 (2269.00, 4676.50)	3540.50 (2871.00, 4614.50)	(-154.00, 681.00)	0.21
	Change (207)	-223.00 (-1149.00, 724.00)	-324.00 (-1179.00, 889.00)	(-519.00, 466.00)	0.89

^a ^Final at 8 weeks follow-up; Change between the final and the baseline measurement

^b ^IQR: Interquartile Range

^c ^p-value based on Wilcoxon 2-group comparison

No significant differences were observed in changes in albumin, and CD4, CD8, and CD3 T-cell counts between the placebo and the supplemented groups over follow-up. However, significant effect modification by age group was observed in CD8 T-cell counts (p = 0.01). Children 3 years and older showed a median increase in CD8 T-cell counts of 135 when receiving multivitamins, compared to a median decrease of 158 observed in children in the same age group receiving placebo (95% CI = 37.00, 1029.00; p = 0.03).

On restricting the analyses to only the 87 HIV-infected children, a similar relationship was observed for hemoglobin as in the overall cohort. Children in the supplemented group gained a median of 1.2 g/dL of hemoglobin compared to no change in the median of the placebo group (p < 0.01).

## Discussion

We found that daily multivitamin supplementation in children with TB in a resource-limited setting resulted in an improvement in hemoglobin levels after two months of follow-up in all age groups and irrespective of HIV status. However, there was no effect of the supplement on albumin levels and growth indices, including weight, length/height, mid-upper arm circumference, head circumference, and triceps skin-fold thickness in the overall cohort. In subgroup analyses, significant weight gain among the youngest children (six weeks to six months) was observed. The results were similar in children also co-infected with HIV; however, these children had a significantly higher increase in height if between the ages of 6 months and three years.

There have been no earlier studies of multivitamin supplementation among children with TB. However, similar beneficial effects of supplements on hemoglobin levels have been obtained with micronutrient supplementation in children in other parts of the world. A recent review by Allen *et al *reported that multiple micronutrient supplementation leads to a significant increase in hemoglobin in children (effect size 0.39; 95% CI 0.25, 0.53) [26]. A pooled analysis used data from intervention trials in Indonesia, Peru, South Africa, and Vietnam among 1134 infants who had been randomized to either a placebo, weekly multiple micronutrient supplement (including vitamins A, D, E, K, C, B-1, B-2, B-6, and B-12, niacin, folate, iron, zinc, copper, and iodine), daily multiple micronutrient supplement, or daily iron supplements. The daily micronutrient supplement was found to be the most effective in controlling anemia and iron deficiency [27].

The results are biologically plausible since the vitamins included in the supplement may lead to better hematologic status through several mechanisms [28]. For example, vitamin C improves intestinal absorption of iron and may also enhance mobilization of iron stores and riboflavin is necessary for the synthesis of the globin component of hemoglobin.

The effects on growth indices have comparatively been less consistent; the review by Allen *et al *reported small yet statistically significant improvements in length/height and weight in children supplemented with multiple micronutrients [26]. On the other hand, the pooled analysis cited above found that infants receiving a daily micronutrient supplement had significantly greater weight gain, whereas there were no differences in height gain [27]. In another meta-analyses of effects of micronutrient interventions on growth of children under five years of age, Ramakrishnan *et al*. found that multiple micronutrient interventions improve linear growth only and had no effect on weight gain [18]. Additionally, a few studies of multiple micronutrient supplementation in adults with tuberculosis have also been equivocal in their results on weight gain. For example, a study in Mwanza, Tanzania, found that multiple micronutrient supplementation (vitamins A, B-complex, C, D, and E, selenium, copper, and zinc) for the first two months of TB treatment led to reduced weight gain among the HIV-infected TB patients; the HIV-uninfected TB patients demonstrated a non-significant increase in weight at the end of follow-up [29].

Several studies have found that serum albumin is lower among patients with TB [13,14,30-35]; however, there are no studies assessing the effect of multivitamin supplementation on albumin levels among children with TB that we can directly compare our results to. The increase in albumin in all children observed in this trial is probably a response to adequate treatment for TB.

The main limitations of our trial were the small sample size and a short period of supplementation and follow-up; this could have led us to miss a beneficial effect of multivitamins on growth indices in the overall cohort, the primary outcome. The trial also was not designed to measure effects of multivitamins in subgroups such as those defined by age or HIV status; therefore, the findings of weight gain among the youngest children or height differences among HIV infected children between the ages of 6 months and 3 years cannot be treated as conclusive. Further, it is possible that we may have included children with other diseases, as TB diagnosis was not optimal. It is also possible that the nutrients such as zinc and vitamin D that were not included in our supplement are more essential for growth. The results of this trial should be generalizable to children with TB, with or without HIV co-infection, in most resource-limited settings.

The multivitamin supplement that we used has the potential to have several beneficial effects including on immune function in growing children, particularly those with TB as they may have several underlying micronutrient deficiencies, including those of vitamins B-complex, C, and E [36]. These nutrients are extensively involved in the immune system and its ability to fight infectious diseases such as TB. For example, the B-vitamins are involved in increasing lymphocyte production, cell-mediated cytotoxicity, delayed-type hypersensitivity responses, and antibody production [37,38]. Vitamin C helps improve T- and B-lymphocyte proliferative responses and reduces the concentration of proinflammatory cytokines [39-41]. Vitamin E is responsible for improving delayed type hypersensitivity skin response, increasing IL-2 production, neutrophil phagocytosis, lymphocyte proliferation, and antibody response to T-cell dependent vaccines, and reducing production of inflammatory cytokines such as TNF- α and IL-6 [42,43]. However, we did not observe an association between immune markers such as CD4, CD8, and CD3 T-cell count with multivitamin supplementation, except for children older than 3 years of age. We are not aware of any known age-specific effects of vitamins on CD8 cells in this age group that may explain this finding.

In conclusion, multivitamin supplementation had no effect on weight gain, the primary outcome of the trial. However, supplementation, even for a short period of eight weeks, improved the hematological profile of all children with TB and led to significant weight gain amongst the youngest patients (n = 22; age six weeks to six months). It is possible that older children need even greater doses of such nutrients to demonstrate an effect and for longer periods of time. The impact of multivitamin supplementation on other parameters such as treatment outcomes needs to be assessed in larger trials with a longer period of supplementation. If proven to be efficacious, multivitamin supplementation could represent an inexpensive adjunct to anti-tuberculous therapy, particularly in resource-limited settings.
